# Highlighting the Mechanistic Relationship Between Perinatal Depression and Preeclampsia: A Scoping Review

**DOI:** 10.1089/whr.2022.0062

**Published:** 2022-10-26

**Authors:** Mei Yuan, Samantha Bedell, Barbra de Vrijer, Genevieve Eastabrook, Jefferson C. Frisbee, Stephanie J. Frisbee

**Affiliations:** ^1^ Department of Pathology and Laboratory Medicine, Department of Obstetrics and Gynaecology, Schulich School of Medicine and Dentistry, University of Western Ontario, London, Canada.; ^2^ Maternal-Fetal Medicine, Department of Obstetrics and Gynaecology, Schulich School of Medicine and Dentistry, University of Western Ontario, London, Canada.; ^3^ Lawson Health Research Institute, London, Canada.; ^4^ Department of Medical Biophysics, Schulich School of Medicine and Dentistry, University of Western Ontario, London, Canada.; ^5^ Department of Epidemiology and Biostatistics, Schulich School of Medicine and Dentistry, University of Western Ontario, London, Canada.

**Keywords:** pregnancy, antenatal depression, depressive symptoms, preeclampsia, gestational hypertension, postpartum depression, cardiovascular disease risk factors

## Abstract

**Background:**

Although there is scientific literature supporting an association between depression and preeclampsia (PE), little is known about the underlying mechanistic pathways that may explain these observed associations. Thus, this study aimed to outline the relationship between depression and PE, and to highlight the underlying cardiovascular and metabolic risk factors that are common to both.

**Methods:**

A scoping review of the literature was conducted in Medline, Scopus, and Web of Science.

**Results:**

From 706 articles initially identified, 23 articles met the inclusion criteria and were included in this review. Although some studies reported a positive association between PE and postpartum depressive symptoms, challenges comparing different methodologies, measurement instruments and when measurements were administered, and patient populations do not permit a decisive conclusion. In addition, very few studies addressed potential underlying mechanisms that may be contributing to observed associations; thus, a secondary search was conducted to identify cardiovascular and metabolic risk factors that are common to both depression and PE.

**Conclusion:**

The cardiovascular and metabolic risk factors (*i.e.,* increased inflammation and oxidative stress and decreased vascular and endothelial function) common to both depression and PE suggest that these factors may contribute as underlying mechanisms in both conditions. These similarities underscore the importance to better understand these mechanisms so preventative and therapeutic strategies could be developed to improve maternal health.

## Background

Hypertensive disorders of pregnancy are a broad spectrum of conditions ranging from chronic hypertension to preeclampsia (PE) and eclampsia. In 2018, ∼7% of all pregnancies in Canada were complicated by hypertensive disorders of pregnancy.^1^ PE is defined as having hypertension (systolic blood pressure ≥140 mmHg and/or diastolic blood pressure ≥90 mmHg), certain end-organ dysfunction, and with or without new-onset proteinuria.^1,2^ PE can lead to health consequences such as long-term end-organ dysfunction, fetal developmental issues, and even death for both mother and fetus.^2,3^ Known risk factors for PE include age, nulliparity, chronic hypertension, body mass index (BMI) >30 kg/m^2^, and preexisting diabetes.^4^ Of concern is evidence from epidemiologic studies demonstrating the increased prevalence of these risk factors in women of childbearing age.^5,6^ In Ontario, between 2012 and 2016, 17.8% of mothers with live singleton or twin births had a prepregnancy BMI >30 kg/m^2^, and 1.1% had established chronic hypertension.^7^ Average maternal age has also steadily risen since the mid-1960s, with the average maternal age at first pregnancy now at 30.8 years.^8^ Additional factors presenting challenges to pregnant women are mental health concerns, particularly depression and depressive symptoms. In Canada, ∼5% of women have major depressive disorder,^9^ in Ontario, 7.7% of mothers were affected with depression during pregnancy,^10^ 8.6% of women experience postpartum depressive symptoms,^11^ and 54.2% of women who experienced postpartum depression had depression previously.^10,12^

Increasing attention is being paid to the interrelationships between preexisting mental health concerns, particularly depression, obesity, and PE. For example, one study reported that, in pregnant women, a one unit increase in prepregnancy BMI was associated with ∼3% higher odds of antenatal depression.^13^ These findings suggest an interplay between physical and mental health, or risk factors that are similar for both entities.

Despite known associations between depression and PE, much less is understood about the possible mechanistic pathways connecting these two conditions. Thus, the first objective for this study was to conduct a scoping review to summarize research findings regarding the association between depression and PE. The secondary objective was to extract information from previously reported studies, particularly related to underlying cardiovascular and metabolic risk factors, and integrate this information with the goal to provide evidence for and insights into the possible mechanistic pathways connecting depression and PE.

## Methods

### Identifying the research question

This scoping review was conducted using the methodological framework described by Arksey and O'Malley.^14^ The primary research question for this scoping review was, “what is the current evidence for an association between depression and preeclampsia”? As most of the screening tools used to assess depression during pregnancy within research are solely to identify those in need of further clinical assessment, the distinction between depressive symptoms and clinical diagnosis of depression is not often made, thus for this scoping review, the term “depression” includes the entire spectrum of the disease. The secondary research question for this scoping review was, “what information has been reported that provides insight into pathophysiologic mechanisms that might link depression and preeclampsia”?

### Identifying relevant studies

This scoping review identified, retrieved, and evaluated information from peer-reviewed scientific articles that examined the association between depression and PE in pregnant women. The focus was on studies published between 2000 and 2020, as it reflected the most recently published literature. Two authors performed the search using a combination of search terms related to our target group, depression, and PE (Table 1) within the following international databases: Medline (2000–April 2020), Scopus (2000–2019), and Web of Science (2000–2020). The search strategies were limited to scientific journal articles and English language only.

**Table 1.** Keywords and Search Terms Used in the Database Searches

Target group or population	Issue	Outcomes
Pregnant	Depression	PE
Pregnancy	Depressive symptoms	
	Major depressive symptoms	
	Prenatal depression	
	Antenatal depression	
	Postpartum depression	

PE, preeclampsia.

### Selecting the literature

#### Inclusion and exclusion criteria

Inclusion criteria for studies included in this scoping review were as follows: (1) an examination of the associations between depression and PE during pregnancy and/or postpartum, and (2) measurement of maternal depression and PE during pregnancy and/or postpartum. Studies were excluded if: (1) depression was pooled with other variables, such as anxiety, stress, and mood disorder, or the PE measurement was pooled with perinatal outcomes, and (2) if the study did not separately assess depression without antidepressant medication. The exclusion of these articles allowed for a more focused examination of the relationships between depression and PE, as covariates such as antidepressant medication have been reported to affect either depression or PE. The specific inclusion and exclusion criteria are given in Table 2. As we included different types of studies in this scoping review (primary studies and review studies), and there is not a single approach to compare the quality of these different types of studies, we did not include a formal assessment of the methodological quality of the included studies. However, we include in the summary table multiple characteristics of the study, including sample size and design, for readers to review and consider.

**Table 2.** Inclusion and Exclusion Search Terms in All Three Databases

Inclusion	Exclusion
Journal articles	Neonatal outcomes
English language	Comorbidities (preexisting disease of liver, kidney, autoimmune disease, *etc.*)
Human population	Drug
Direct measurement of depressive symptoms/PE	Pharmaceutical
	Therapy
	Treatment

**FIG. 1.**
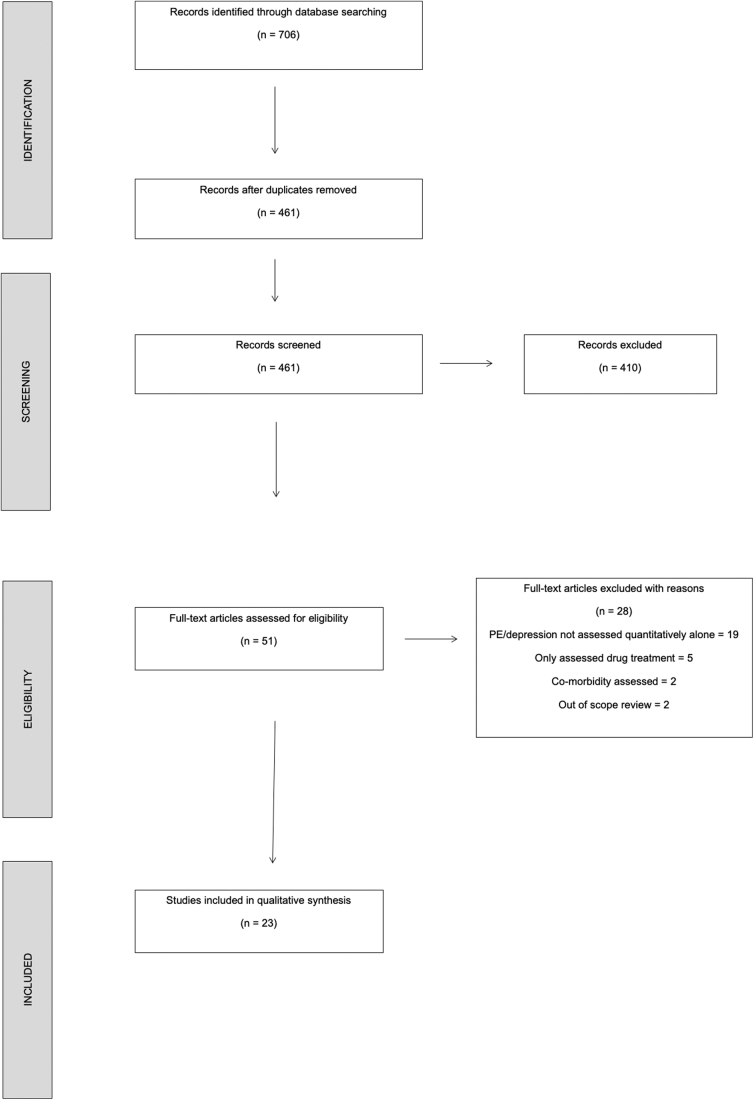
PRISMA-Sr search strategy. This flowchart displays the search process from identification of relevant literature to final inclusion of full test articles.

Duplicate articles were identified by the search tool and removed from the database before screening. A two-step process was performed by two authors (M.Y. and S.F.) (Fig. 1). During the first stage, the titles and abstracts of the identified articles were screened according to the exclusion and inclusion criteria described previously. Uncertainty during the initial title and abstract screening was resolved by consulting the full-text articles.

#### Abstracting information and summarizing data from included studies

Studies that met the inclusion criteria were screened further as full articles. Full-text screening was assessed for final inclusion into the study, with the selected full-text articles passing an additional bibliographic reference list check to identify any additional eligible publications. The articles reviewed were categorized and summarized depending upon whether the study assessed the outcomes of interest during prenatal period (before the birth of the newborn) or postpartum period (after delivery of the newborn). Information such as the author, year of publication, study design, study population, outcomes, relevant findings, and information about underlying cardiovascular or metabolic risk factors examined was also abstracted.

### Supplemental literature search

To better interpret and supplement the information abstracted from studies included in this scoping review related to the secondary research question (pathophysiologic mechanisms that might link depression and PE), a supplemental literature search was conducted. This general search, conducted in PubMed, identified research studies with information that could better explain information regarding the underlying mechanisms of depression and PE obtained from studies included in this scoping review.

## Results

Twenty-three articles were included in this scoping review. Nine studies were conducted in North America, six in Europe, five in Asia, two in South America, and one in Africa. Four meta-analyses were included as they fit the inclusion criteria in addressing the research question, despite having a few studies within the meta-analyses that overlapped with those already included within the review. Twelve studies were published in the most recent 5 years.

### Associations between prenatal depression and PE during pregnancy

Of all 23 articles reviewed, 13 explored the associations between prenatal depression and PE during pregnancy (Table 3). Some studies reported a significant positive association between the two factors,^15–20^ but other studies did not observe such associations or reported associations that were not statistically significant.^21–23^ In a prospective case–control study carried out in Iran, Kharaghani et al observed that women with mild depression and severe to moderate depression had a 1.81-fold and 2.52-fold increased risk of developing PE, respectively.^17^ Conversely, Ghaffar et al observed no significant association in hospitalized women between depression and PE.^21^ Vollebregt et al also reported no significant associations between depression and PE even after adjusting for medical and socio-environmental factors.^23^

**Table 3.** Summary and Synthesis of Primary Studies Examining the Relationship Between Prenatal Depressive Symptoms and Preeclampsia

First author and year	Country	Study design	Study population and sample size	Measurements/assessment tools	Outcome	Relevant findings	Examination of potential underlying factors
Bansil et al (2010)^16^	United States	Retrospective cross-sectional	In-hospital delivery visits from 1998 to 2005 nationwide inpatient sample of women aged from 15 to 44; *n* = 32,156,438	ICD-9 codes for concurrent diagnosis of depression and pregnancy complications, including PE	PE	1.57 risk of PE (OR, 95% CI, 1.52–1.62) in women with depression; rate of depression increased during the 7-year study period (2.73/1,000 to 14.12/1,000 deliveries in 2005)	Not examined in this study
Ghaffar et al (2016)^21^	Pakistan	Prospective cross-sectional	Pregnant women 16–40 years old at >12 weeks' gestation, presenting with obstetric risk to a tertiary hospital; *n* = 230	EPDS administered at mid-pregnancy; participants classified as depressed or not depressed (score >11)	Hypertension, PE, eclampsia, and other pregnancy complications	60% of participants were classified as depressed. Significant statistical associations between depression and hypertension, but no statistically significant associations reported between depression and PE, eclampsia, or other pregnancy complications	Hypertension associated with depression
Kharaghani et al (2012)^17,a^	Iran	Prospective case–control	All new singleton admission to the hospitals around 35.7 weeks, *n* = 156 with PE, 156 pregnant with healthy pregnancy	PHQ-9 at enrolment (mean gestational age was 35.6 weeks)	PE	Moderate-to-severe depression observed in 31.2% of the PE group (24.8% in control). Statistically significant associations observed in trend test for severity of depressive symptoms and PE (statistically significant mild depression had 1.81 (OR, 95% CI, 1.05–3.14)-fold increase risk of PE; severe to moderate depression had a 2.52 (1.05–6.02)-fold increase)	Associations between depression and PE remained statistically significant after adjusting for prepregnancy BMI, but magnitude of statistical significance of BMI itself was not reported
Kim et al (2013)^20,a^	United States	Retrospective cohort	Analyzed data from black, non-Hispanic pregnant women presenting for initial prenatal visit, with singleton birth past 20 weeks gestational age; *n* = 261	EPDS at initial pregnant visit; mean gestational age for EPDS screening was 17.2 weeks	PE, IUGR, PTB	An EPDS score^3^ 10 was statistically significantly risk factor for PE (OR = 2.95, 95% CI, 1.26–6.89), PTB, and IUGR; 9.6% of the women had PE	Not examined in this study
Kurki et al (2000)^15,a^	Finland	Prospective cross-sectional	Pregnant women enrolled at first prenatal visit between 8 and 17 weeks' gestation; specific exclusion for elevated risk of PE, essential hypertension, GDM, and twins; *n* = 623	BDI conducted at median 12 weeks' of gestation	PE	Depression associated with 2.5-fold increased for PE (95% CI, 1.2–5.3)	Not examined in this study
Palmsten et al (2012)^22^	Canada	Prospective cohort	Live birth pregnancies with continuous health care enrolment until 2 months after delivery; at least one inpatient or outpatient code for depression during year before LMP; *n* = 306,831 pregnancies from 224,827 women	ICD-9 codes for PE and depression diagnosis	PE	No statistically significant results reported as the risk of PE was similar in women without depression and women with depression, but no antidepressant dispensing (2.3% vs. 2.4%)	Not examined in this study
Qiu et al (2007)^18,a^	Peru	Prospective case–control	Cases: women with PE diagnosis at 20 weeks' gestation; controls: women with uncomplicated pregnancies; *n* = 339 case and 337 control	PHQ-9 during pregnancy	PE	Moderate depression (score 10–14) during pregnancy had 2.6-fold increase risk of PE (OR = 2.6; 95% CI, 1.4–4.6); moderate-to-severe depression (score, 15–19) was associated with 3.2-fold increase risk (OR = 95% CI, 1.1–9.6) of PE after adjusting for confounders	Associations between depression and PE remained statistically significant after adjusting for prepregnancy BMI, but magnitude of statistical significance of BMI itself was not reported
Qiu et al (2009)^19^	United States	Retrospective cohort	Women who initiated prenatal care before 20 weeks gestation, older than 18 years old, delivered at the required hospital; *n* = 2,601	Depression and PE diagnosis based on medical records (could be ICD or just self-report for depression); data analyzed women enrolled between 1996 and 2004	PE	Disorders diagnosed during the first 20 weeks of pregnancy were more strongly related with PE risk (RR = 3.64; 95% CI, 1.13–11.86); women classified as having any mood or anxiety disorder had 2.12-fold increased risk of PE (RR = 2.12, (1.02–4.45); women with isolated mood disorder had 2.72-fold increased PE risk compared to women without psychiatric disorders (95% CI, 1.29–5.74.	Prepregnancy BMI, family history of hypertension, age, and diabetes were evaluated to see if it modified the effect of maternal mood disorders and PE risk, but no significant modifications were observed
Thombre et al (2015)^34^	United States	Prospective case–control	Pregnancy women over age of 15, singleton pregnancy with no known congenital or chromosomal abnormalities or pregnancy diabetes between 16 and 27 weeks of gestation; *n* = 1,371	Depression and anxiety symptoms: history taken at four time points; For depression within the past week, CES-D (score of 16 and greater is depressed)	PE	Prepregnancy history of depression symptoms was associated with presence of a hypertensive disorder during pregnancy (OR = 1.8; 95% CI, 1.1–3.2) à driven by women with chronic hypertension	Associations between depression and PE remained statistically significant after adjusting for prepregnancy BMI and smoking, but magnitude of statistical significance of BMI and smoking was not reported
Vollebregt et al (2008)^23,a^	The Netherlands	Prospective cohort	Nulliparous women with singleton pregnancy who attendee antenatal care for first check-up and completed the questionnaire before 24 weeks and delivered after 24 weeks; *n* = 3,679	Psychosocial stress: work stress, depression (Dutch CES-D), anxiety (Dutch STAI), and pregnancy-related anxiety (revised version of Pregnancy-Related Anxiety Questionnaire	PE	No association observed between anxiety, depression, or pregnancy related anxiety and PE and GH after adjusting for medical and socioeconomic covariates	Associations between depression and PE remained statistically significant after adjusting for prepregnancy BMI, but magnitude of statistical significance of BMI itself was not reported
*Synthesis and summary of table*			*10 of the 23 studies that met the inclusion criteria for this review. Seven of the 10 studies reported statistically significant associations between prenatal depressive symptoms and PE (PE)*				

^a^ Studies included in the meta-analyses are given in Table 4.

BDI, Beck Depression Inventory; BMI, body mass index; CES-D, Center for Epidemiological Studies- Depression; CI, confidence interval; EPDS, Edinburgh Postpartum Depression Scale; GDM, gestational diabetes mellitus; GH, gestational hypertension; ICD-9, International Classification of Diseases, 9th Revision; IUGR, Intrauterine growth restriction; LMP, last menstrual period; OR, odds ratio; PHQ-9, Patient Health Questionnaire-9; PTB, pre-term birth; RR, relative risk.

Pooled analyses from two meta-analyses, Hu et al (pooled odds ratio [OR] = 1.48; 95% confidence interval [CI], 1.04–2.01) and Zhang et al (pooled OR = 1.5; 95% CI, 1.10–20.5) reported statistically significant positive associations between prenatal depressive symptoms and PE.^24,25^ Conversely, Grigoriadis et al did not observe any statistically significant associations between exposure to antenatal depressive symptoms and an increased risk of PE (OR = 1.35, 95% CI, 0.95–1.92).^12^ Summaries and details of these three meta-analyses are given in Table 4.

**Table 4.** Summary and Synthesis of Meta-Analyses Examining the Relationship Between Prenatal Depressive Symptoms Preeclampsia

First author and year	Country	Aim	Studies included within our search	Outcome	Statistical findings	Examination of potential underlying factors
Grigoriadis et al (2013)^12^	Canada	Whether maternal depression during pregnancy is associated with perinatal and infant outcomes	Kurki et al,^15^ Vollebregt et al^23^	PE, perinatal outcomes, infant outcomes (searched Medline, PsycInfo, CINAHL, Embase, Scopus); *n* = 30	Association between exposure to maternal depression and PE was not statistically significant from pooled OR of 4 studies (1.35, 95% CI, 0.95–1.92)	Not examined in this study
Hu et al (2015)^24^	United States	Whether antenatal depression is a risk factor for PE or operative deliveries	Kharaghani et al,^17^ Kim et al,^20^ Kurki et al,^15^ Qiu et al,^18^ Vollebregt et al^23^	PE, operative deliveries (searched through PubMed, SCI/SSCI, Proquest PsycArticles, CINAHL); *n* = 12	Pooled OR of 1.63 times (95% CI, 1.32–2.02) increased risk for PE in patients with antenatal depressive symptoms (after adjusting for prepregnancy BMI, (OR = 1.48, 95% CI, 1.04–2.10)	Associations between depression and PE remained statistically significant after adjusting for prepregnancy BMI, but magnitude of statistical significance of BMI itself was not reported
Zhang et al (2013)^25^	China	Whether mental stress affects gestational hypertension or PE	Kharaghani et al,^17^ Kurki et al,^15^ Qiu et al,^18^ Vollebregt et al^23^	PE, gestational hypertension (PubMed, Cochrane, Chinese medical datasets); *n* = 13	5 cohort studies, 8 case–control, pooled effect of the 12 studies suggested that mental stress was associated with an increased risk of PE (OR = 1.49; 95% CI, 1.27–1.74, *p* < 0.001); depression alone was observed to have statistically significant associations with PE (OR = 1.5, 95% CI, 1.10–2.05) and no heterogeneity. Overall, the effect size in PE was larger than GH	Not examined in this study
*Synthesis and summary of the table*			*3 of the 23 studies met the inclusion criteria for this review. Two of the three meta-analyses reported statistically significant associations between prenatal depression and PE*			

### Associations between PE and postpartum depression

Ten of the 23 articles included in this scoping review investigated associations between diagnosis of PE and development of postpartum depression (Table 5). Seven studies reported significant associations between increased prevalence of postpartum depression with the diagnosis of PE.^26–32^ Auger et al observed that women with PE had a higher incidence of hospitalization for depression 28 years after indexed delivery and that late-onset PE was more consistently associated with hospitalization for depression within 1–4 years after pregnancy.^30^ Similarly, Bergink et al reported an increased incidence risk ratio of 2.85 (95% CI, 1.84–4.42) for unipolar depression within 0–3 months postpartum in women who developed PE.^31^

**Table 5.** Summary and Synthesis of Primary Studies Examining the Relationship Between Preeclampsia and Postpartum Depressive Symptoms

First author and year	Country	Study design	Study population and sample size	Measurement/assessment tools	Outcome	Relevant findings	Examination of potential underlying factors
Auger et al (2021)^30^	Canada	Longitudinal cohort	Women at 20+ weeks' gestation; diagnosed PE; followed for 28 years; *n* = 1,210,963	ICD-9 and ICD 10 classification of PE	Incidence of depression hospitalization per 1,000 person-years (calculated using cumulative incidence function) as proxy for severe depression	In adjusted models, late-onset PE was statistically significant associated with increased risk of depression hospitalization later in life. Associations were present for 28 cumulative years, 1–4 years, 4–14 years, and ≥15 years after pregnancy	Associations between PE and depression remained statistically significant after adjusting for obesity, GDM, and diabetes but magnitudes of statistical significance was not reported
Bergink et al (2015)^31,a^	Denmark	Retrospective cohort	Singleton and primiparous women born in Denmark, pregnant between 1995 and 2011; *n* = 400,717	Danish National Patient/Hospital Register, ICD-10 diagnosis of PE	Depression (ICD-10), based on first contact with Danish Psychiatric Central Research Register after delivery	All pregnant women were at increased risk of experiencing first-onset psychiatric episodes 0–3 months postpartum, with a considerable increased risk for women with PE	Associations between PE and depression remained statistically significant after adjusting for gestational diabetes mellitus was measured but magnitude of statistical significance was not reported
Chen et al (2019)^26^	China	Retrospective cross-sectional	Women aged 20–40 years old, selected from specialty postpartum clinic at 6-week visit, *n* = 180, 90 with PE, and 90 randomly selected non-PE controls	EPDS (score ≥10 for possible depression) at 6 weeks postpartum	Depression	Women with PE had 2.75-fold increase odds of PPD (95% CI, 1.056–7.18); severe PE diagnosis associated with 4.5-fold increase odds of PPD (95% CI, 1.94–17.255)	Associations between PE and depression remained statistically significant after adjusting for predelivery BMI, but magnitude of statistical significance of BMI itself was not reported
Gaugler-Senden et al (2012)^81,a^	The Netherlands	Retrospective cohort	Women diagnosed with severe PE before 24 weeks' gestation and all women with severe PE at 24–32 weeks' gestation admitted and delivered before 34 weeks’; *n* = 104 severe PE and 78 control participants (with spontaneous preterm delivery, matched for age, parity, gestational age at delivery, ethnicity, and year of delivery)	Zung Depression Scale, Impact of Event scale, and Social Readjustment Rating scale	Women with severe PE between 1999 and 2004 were identified and asked to complete surveys in 2008. 79% response rate for PE group and 58% for control group	No observed difference in current and recalled postpartum depression scores in women with severe PE and controls with preterm delivery up to 7 years; women with induced PE observed to have higher posttraumatic score	Not examined in this study
Hoedjes et al (2011)^32^	The Netherlands	Prospective cohort	Participants drawn from women enrolled in the Pro-Active study; gave birth between February 2007 and June 2009, over the age of 18, had PE, and understood Dutch; *n* = 174	EPDS at 6, 12, and 26 weeks postpartum, cutoff score of 10 for possible depression	Depression	Women with severe PE (ACOG criteria) group were more likely to report depressive symptoms postpartum, anytime up to 26 weeks compared with women with mild PE (44% vs. 23%); perinatal death and NICU admission were reported to be contributing factors	Not examined in this study
Mbarak et al (2019)^27^	Tanzania	Prospective cross-sectional	Postpartum women diagnosed with PE or eclampsia (as abstracted from discharge notes) delivered and attended postnatal clinic at MNH, enrolled before 4 weeks postpartum; *n* = 386	EPDS at 2–6 weeks postpartum, score >13 considered as depression	Depression	Younger women diagnosed with PE or eclampsia was more likely to suffer from PPD (AOR = 10.13; 95% CI, 1.99–52.02); only 10.5% of women with mild PE had PPD, compared with 25.3% and 31.7% of women with severe PE and eclampsia, respectively	Not examined in this study
Mommersteeg et al (2016)^35,a^	The Netherlands	Longitudinal cohort	4 years follow-up of women enrolled in the PREVFEM study (gave birth from 1991 to 2007, participants classified as early-onset PE or no PE based on ISSHP criteria); invited to complete mailed-in surveys in 2013; *n* = 265 in PE group, 268 in age-matched non-PE control group	Depression: PHQ-9 (PHQ-9 score ≥5 indicating mild to severe depressive symptoms), anxiety: GAD-7, fatigue: fatigue assessment scale	Depression	PE group reported significantly more depressive symptoms after adjusting for covariates (*B* = 0.70; 95% CI, 0.09–1.32; *p* = 0.026)	Associations between PE and depression remained statistically significant after adjusting for BMI, but magnitude of statistical significance of BMI itself was not reported
Strapasson et al (2018)^28^	Brazil	Prospective cross-sectional	Convenience sampling of women who had recently delivered, HDP group had to be diagnosed with HDP after 20 weeks, age older than 18 years; *n* = 168 (42 diagnosed with HDP, 126 normotensive)	EPDS (score ≥12 considered depressed) within 12 hours postpartum and medical charts for pregnancy outcomes and HDP diagnosis	Depression	All HDP (severe PE, superimposed PE, eclampsia) were significantly associated with PPD. Probability of postpartum depression correlated with the diagnosis of HDP was significant	Not examined in this study
Youn et al (2017)^29,a^	South Korea	Retrospective cross-sectional	Data extracted from Korean national health insurance system from September 2016 to January 2017 on women who gave birth between January 1, 2010 and December 31, 2012, only included the first pregnancy; *n* = 1,269,130	Classified into previous history of depression (up to 1 year before birth) and depression during the postpartum period (first year after delivery) based on ICD-10 code for depression; presence of PE determined based medical records	Depression included depression before pregnancy and postpartum (up to 1 year)	PE increased risk of PPD (OR = 1.12; 95% CI, 1.03–1.22). Previous history of depression had a higher risk for PPD (OR = 16.72; 95% CI, 16.05–17.41); women who delivered preterm or had placental abruption were more likely to develop PPD	Not examined in this study
*Summary and synthesis*		*9 out of the 23 studies met the inclusion criteria for this review. Eight of the nine studies reported statistically significant associations between PE (PE) and postpartum depressive symptoms.*					

^a^ Studies included in the meta-analysis in Table 6.

ACOG, American College of Obstetrics and Gynecology; AOR, adjusted OR; HDP, hypertensive disorders of pregnancy, HR, hazards ratio; ISSHP, International Society for the Study of Hypertension in Pregnancy; MNH, Muhimbili National Hospital; NICU, neonatal intensive care unit; PPD, postpartum depression.

Caropreso et al was the only meta-analysis that explored the association between PE and postpartum depression in our search. The analysis reported the standard mean difference between groups of women with and without PE as 1.04 (95% CI, 0.22–1.86), which suggested high severity of depressive symptoms in women with a history of PE.^33^ Study details of this meta-analysis are given in Table 6.

**Table 6.** Summary and Synthesis of Meta-Analysis Examining the Relationship Between Preeclampsia and Postpartum Depressive Symptoms

First author and year	Country	Aim	Studies included within our search	Study design	Outcome	Statistical findings	Examination of potential underlying factors
Caropreso et al (2020)^33^	Canada	Whether PE is a risk factor for postpartum depression and psychosis	Bergink et al,^31^ Gaugler-Senden et al (2012),^81^ Mommersteeg et al,^35^ Youn et al^29^	Meta-analysis of 8 of 13 studies reviewed (searched through PubMed, PsycInfo, Scopus, Embase), appraised using Newcastle-Ottawa scale	Depression and psychosis	All 8 studies observed association between PE and postpartum depression or depression outside the perinatal period. Postpartum: standard mean difference of depressive symptom severity between women with and without history of PE was 1.04 (95% CI, 0.22–1.86; *p* = 0.01)	Some studies assessed BMI and GDM but the review itself did not report individual magnitudes of effect on these variables in associations with PE and PPD

This table includes 1 of the 23 studies that met the inclusion criteria for this review and indicated high severity of postpartum depressive symptoms in women with a history of PE.

### Examination of common underlying cardiovascular and metabolic risk factors

The majority of the articles included in this scoping review accounted for factors associated with cardiovascular risk, such as prepregnancy BMI, smoking, preexisting hypertension, and diabetes. In the studies investigating associations between prenatal depression and PE, the associations observed between depression and PE remained after adjusting for these cardiovascular risk covariates.^18,19,23,34^ In studies assessing the association between PE and postpartum depression, three of the nine studies adjusted for the confounding variable of prepregnancy BMI. As given in Table 5, all three articles, Auger et al, Mommersteeg et al, and Chen et al, reported that positive associations between PE and postpartum depression remained significant after adjustment for prepregnancy BMI.^26,30,35^

### Integration of information from supplemental literature search

Although all included studies were examined for common underlying cardiovascular and metabolic risk factors, it is important to note that none of the studies reported the magnitude of an independent association between the covariates and the association between depression and PE, or more advanced analyses such as stratification or mediation analysis that might provide insight into the characteristics underlying the observed association between PE and depression. Similarly, none of the studies specifically assessed potential common cardiovascular and metabolic risk factors such as arterial stiffness, endothelial dysfunction, or obesity between depressive symptoms and PE despite previous studies suggesting that these factors are associated with both PE and depression individually.^36–39^ To provide better insight into possible pathophysiologic mechanisms that might link depression and PE, integration of the results from this scoping review and information from a supplemental scoping review focused on BMI, vascular stiffness, endothelial dysfunction, inflammation, and oxidative stress are now presented.

#### Body mass index

A high prepregnancy BMI is associated with an increased risk of antenatal depression.^40^ This is unsurprising as obesity and depression outside of pregnancy are often linked and are thought to share similar biological mechanisms.^41^ In addition, studies have shown that an elevated BMI increases the risk for developing PE^42,43^; although obesity alone is not a direct indicator of cardiometabolic health and recent studies have begun to consider the differences between “healthy” obesity and obesity with associated metabolic abnormalities.^44^

#### Arterial stiffness

Arterial stiffness is a good indicator of cardiovascular health and common measurements of arterial stiffness include augmentation index, which represents arterial adaptability, and pulse-wave velocity. In the nonpregnant literature, depression has been associated with a higher augmentation index and pulse wave velocity measurements,^37,45–47^ suggesting that individuals with depression have increased arterial stiffness that may contribute to increased cardiovascular risk. Similarly, multiple studies have reported that individuals with a current and/or previous history of PE have significantly higher pulse wave velocity and augmentation indices compared with those who had healthy, uncomplicated pregnancies.^46,48–56^

#### Endothelial dysfunction

Impaired measurements of flow-mediated dilation, an ultrasound-based imaging assessment of dilation in the brachial artery that is used to quantify the health or dysfunction of the endothelium, have been associated with depression in the nonpregnant population.^57,58^ Prenatal measurements of flow-mediated dilation have been reported to be impaired in pregnant women with established PE or those who develop PE later in pregnancy when compared with women with normotensive pregnancies.^49,50,59–61^

#### Inflammation

Inflammation has been implicated in both depression and PE. Studies have found increased levels of inflammatory markers to be associated with both depression and PE. For example, outside of pregnancy, commonly studied inflammatory markers, such as interleukin-1, interleukin-6, and C-reactive protein, have been shown to be positively associated with depression.^62^ In pregnancy specifically, individuals with a history of depression have increased plasma C-reactive protein and glycoprotein acetyls.^63^ Similarly, increased levels of these markers have been shown to be associated with PE.^64,65^

#### Oxidative stress

Markers of oxidative stress have also been studied in both depression and PE. In the general population, depression has been shown to be associated with increased oxidative stress and reduced antioxidant activity.^66^ Furthermore, one study found that antenatal depression was significantly associated with increased levels of 8-isoprostane, a specific biomarker of oxidative stress.^67^ Likewise, individuals with PE have elevated markers of oxidative stress, including 8-isoprostane as well as malondialdehyde and prostaglandin F2α.^64,68^

The well-established relationships between inflammation, oxidative stress, and vascular function and the risk factors associated with both depression and PE (*i.e.,* obesity and hypertension)^55,69,70^ suggest that these factors may contribute as underlying risk factors between depression and PE. These similarities underscore the importance to better understand these mechanisms, so preventative and therapeutic strategies could be developed to improve maternal health.

## Discussion

The scoping review addressing the first objective of this study summarized research findings regarding the association between depression and PE and found conflicting results, although these inconsistent results may reflect the challenges of “standardizing” evaluations during a complex time period. Contextually, pregnancy can span from conception until the delivery of the newborn, suggesting various time points where depressive symptoms can be assessed (*i.e.,* 1st, 2nd, and 3rd trimester). Fluctuating moods and emotions between different stages of the pregnancy may lead to varying levels of negative affect and thus rendering some associations significant and others insignificant. For this reason, the prevalence of depression in pregnancy may be over- or underestimated, depending on the timing of assessment.

The definition of the postpartum period varied from immediately postdelivery, 1–6 months after delivery, and up to several years after the indexed delivery. Despite the varying time of assessment, the majority of the studies reported that there was an increased risk of experiencing postpartum depression following PE. These findings aligned with the meta-analysis performed by Caropreso et al,^33^ which reported that 8 of 13 studies observed associations between PE and postpartum depression or depression outside the perinatal period.

**FIG. 2.**
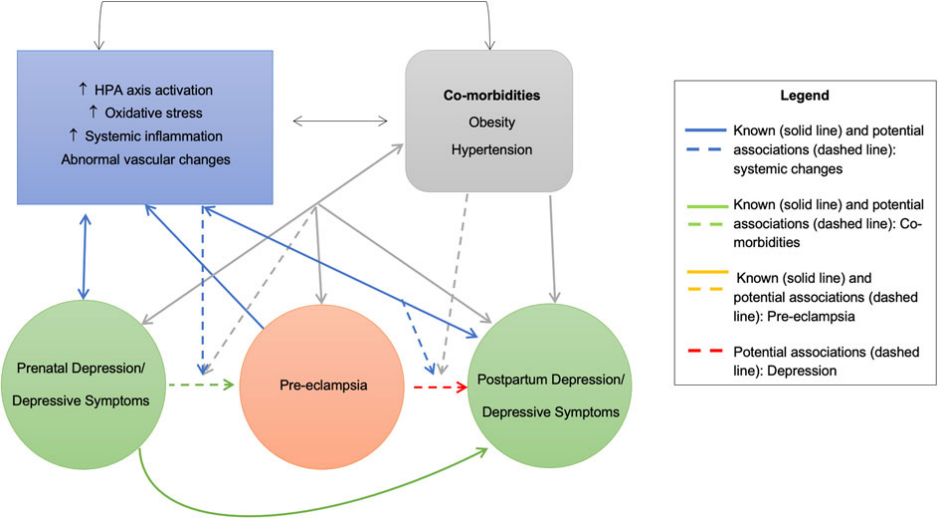
Concept map of associations between risk factors and conditions. Solid lines represent explored and known associations. Dash lines represent relationships of interest for this scoping review: (1) primary interest (orange and red) and (2) potential underlying mechanism (blue and green).

As most of the studies included in our scoping review were epidemiological and thus only assessed the prevalence and association of these disorders in pregnant women, following a secondary literature search, we outlined some of the cardiovascular and metabolic factors associated with both pathologies (*e.g.,* preexisting obesity, increased systemic inflammation, oxidative stress, vascular changes, and endothelial dysfunction) that may contribute to the common underlying etiologies between depression and PE. The complex relationship between these pathologies and risk factors are given in Figure 2.

Many of these risk factors interact with one another and may be linked in a vicious cycle affecting overall cardiometabolic health. For example, both PE and depression are associated with increased rates of obesity and cardiovascular disease, which too share many of these underlying risk factors.^41,44,71–73^ There is substantial evidence from both animal and human studies that depression and cardiovascular disease are linked through changes in oxidative stress, hyperactivation of the hypothalamic–pituitary–adrenal axis and the immune system.^71,74–76^ These changes are also considered mechanistic links between depression and obesity.^41^ PE has been associated with an imbalance between the antioxidant defense system and the production of reactive oxygen species, which then leads to an increase in generalized inflammatory processes and endothelial dysfunction.^77,78^ Endothelial dysfunction in women with PE has also been associated with significantly higher levels of oxidative stress, markers of systemic inflammation, and overactivation of the hypothalamic–pituitary–adrenal axis.^79,80^

Risk factors such as vascular endothelial dysfunction, as evidenced by reductions to the flow-mediated dilation response, are tightly linked with prooxidant and proinflammatory environments across a wide of array of conditions. The “positive feedback loop” that many investigators have identified in linking elevated oxidant stress with chronic inflammation can have the impact of reducing mediators of vascular function such as nitric oxide and prostacyclin bioavailability and increase vascular production of constrictor metabolites such as thromboxane A_2_ among other metabolites of arachidonic acid. Each of these potential contributors may act to increase vascular resistance within human subjects and alter patterns of cerebral perfusion—which has been identified as a likely contributor to cognitive impairments including depressive symptoms.

In addition, reductions to arterial and vascular wall distensibility will have significant impacts on the penetrance of pulse wave hemodynamics deeper into the cerebrovascular circulation and has been implicated in cerebrovascular network remodeling impairing mass transport and exchange within the microcirculation. Taken together, this constellation of vascular impairments and elevated risk factor profiles may represent a significant contributor to the emergence of depressive symptoms, potentially of vascular origin. This is an area of investigation that will require significantly more study in the coming years to better understand how to identify and support women who may be at higher risk for these conditions and help alleviate subsequent complications.

Limitations of this scoping review include restricting to English-only articles from three databases and including all eligible studies without evaluation of the quality or size of the study. In addition, to specifically assess the association between depression during pregnancy and PE, this review excluded any studies that evaluated the possible mediating effects of antidepressant medications. Strengths of this study include the differentiation between prenatal and postpartum depression and the abstraction and synthesis of information related to potential underlying causal mechanisms. Previous reviews on this topic have only assessed the relationship between either prenatal depressive symptoms or postpartum depressive symptoms and PE. Furthermore, this scoping review focused on identifying common underlying cardiovascular risk factors that may contribute to the relationship between the two conditions. This is the only scoping review thus far that investigates the available literature on depression and PE throughout the perinatal period with consideration for common underlying risk factors.

## Conclusion

In conclusion, pregnant women with prenatal depressive symptoms may be at increased risk of developing PE and women who developed PE are at increased risk of developing postpartum depressive symptoms. Moreover, this review highlighted the cardiovascular and metabolic changes that may be contributing to the relationship between the two pathologies and may be useful as early pregnancy indicators of those who are at a higher risk for adverse pregnancy outcomes. Although universal screening for prenatal depression and PE is beneficial and are goals for many obstetrical settings, an overall assessment of cardiometabolic health in early pregnancy may be more useful to detect those who are most at risk for depression and/or PE and their associated complications.

## Abbreviations Used

ACOG: American College of Obstetrics and Gynecology
Aix: augmentation index (indices)
AOR: adjusted odds ratio
BDI: beck depression index
BMI: body mass index (kg/m^2^)
CES-D: Centers for Epidemiologic Studies Depression Screener
CI: confidence interval
CVD: cardiovascular disease
DS/D: depressive symptoms/depression
EPDS: Edinburgh Postnatal Depression Scale
FMD: flow-mediated dilation
GDM: gestational diabetes mellitus
GH: gestational hypertension
HDP: hypertensive disorders of pregnancy
HPA: hypothalamic–pituitary axis
HR: hazards ratio
ICD-9: International Classification of Disease, Version 9
ISSHP: International Society for the Study of Hypertension in Pregnancy
IUGR: Intrauterine growth restriction
LMP: last menstrual period
MDD: major depressive disorder
MNH: Muhimbili National Hospital
NICU: neonatal intensive care unit
OR: odds ratio
PE: preeclampsia
PHQ-9: Patient Health Questionnaire-9
PPD: postpartum depression
PTB: pre-term birth
PWV: pulse-wave velocity
RR: relative risk
